# Knowledge, Attitude and Performance Associated with Disaster Preparedness in Iranian Nurses: A Systematic Review and Meta-analysis

**DOI:** 10.22114/ajem.v0i0.204

**Published:** 2019-07-24

**Authors:** Kosar Yousefi, Haleh Adibi Larijani, Mohammad Golitaleb, Ali Sahebi

**Affiliations:** 1. Ilam University of Medical Sciences, Ilam, Iran.; 2. Iranian Red Crescent Society, Tehran, Iran.; 3. Department of Nursing, School of Nursing and Midwifery, Arak University of Medical Sciences, Arak, Iran.; 4. Department of Health in Disasters and Emergencies, School of Public Health and Safety, Shahid Beheshti University of Medical Sciences, Tehran, Iran.

**Keywords:** Attitude, Disasters, Iran, Knowledge, Nurses, Work Performance

## Abstract

**Context:**

Nurses are the largest group of health service providers with a vital role in assisting victims during disasters. They must be therefore appropriately prepared to respond to health requirements in these situations.

**Evidence acquisition:**

Three articles were ultimately extracted from a comprehensive search conducted in the present systematic review and meta-analysis in Google Scholar, SID, Magiran, Scopus, PubMed and Web of Science using English keywords and their Persian equivalents. The I^2^ index was used to evaluate the heterogeneity between the studies, and the analyses were performed in STATA 14.

**Results:**

This review was conducted on 586 nurses working in hospitals. The final meta-analysis included three articles. The matched mean scores of knowledge, attitude and performance concerning disaster preparedness were respectively found to be 43.49 (95% CI: 37.67–49.31, P=0.581, I^2^=0.0%), 68.81 (95% CI: 63.04–70.58, p=0.913, I^2^=0.0%) and 56.19 (95% CI: 53.95–58.42, P=0.001, I^2^=86.2%).

**Conclusion:**

The present findings suggested moderate levels of knowledge and performance and good attitudes associated with preparedness in disasters in the Iranian nurses. These parameters can be therefore improved to desirable levels and the overall preparedness for coping with disasters boosted in nurses by training nurses and performing hospital drills.

## Context

As a common occurrence throughout the world, disasters severely affect communities, families and individuals (3). Healthcare providers with a key role in responding to disasters are involved in all the planning phases of responding to disasters (5). The perfect preparedness of healthcare providers and workers for encountering disasters is crucial given their pivotal role before, during and after these incidents and the growing incidence and health consequences of these events (6). Disaster management can be accomplished if all members of the healthcare team are aware of their duties and work as a team under the supervision of an integrated management system. Research suggests that healthcare managers need to integrate training programs into disaster management to improve the knowledge and practice of nurses (7). Several studies have shown that most nurses lack the knowledge required for preparedness in disasters, and only a few can adequately respond to these events (8). The present study was therefore conducted to evaluate the level of knowledge, attitude and performance associated with preparedness for disasters in Iranian nurses.

## Evidence acquisition

The present systematic review and meta-analysis collected the data through a review of literature with no time limits conducted by searching Google Scholar, SID, Magiran Scopus, PubMed and Web of Science databases using relevant English keywords and their Persian equivalents, including knowledge, attitude, performance, nurses, preparedness, readiness, healthcare providers, practice, function and Iran. Combinations of the keywords were also applied using Boolean operators, i.e. AND and OR. Articles were searched with no time limits up until May 2019. The review was conducted based on the preferred reporting items for systematic review and meta-analysis (PRISMA) guidelines (9). The articles reporting the mean scores of knowledge, attitude and performance associated with disaster preparedness in Iranian nurses were included. Letter to editors, quasi-experimental studies, brief reports and studies performed in other countries or on healthcare personnel other than nurses were excluded. The STROB checklist was used to assess the articles, each of which received a score of 0–44 and was accordingly assigned to the low-score (<16) category, or the moderate-score (16–30) category or the good-score (31–44) category in terms of quality. The articles receiving a score of at least 16 were ultimately included in the meta-analysis (10).

Given that the parameters assessed in the present research included the mean scores of knowledge, attitude and performance associated with disaster preparedness in nurses, the variances were calculated based on a normal distribution and a 95% confidence interval. The weights assigned to a study was inversely proportional to its variance. The scores reported in different studies were highly mismatched given the use of different data collection tools. The mean scores were therefore matched before applying the fixed effects model in the meta-analysis. An I^2^ index of <25%, 25–75% and ≥75% respectively represented a low, moderate and high heterogeneity (10). Meta-regression was also used to investigate the relationships of the levels of knowledge, attitude and performance associated with disaster preparedness in the nurses with the article’s year of publication. The data collected were analyzed in STATA-14.

## Results

**Figure 1:**
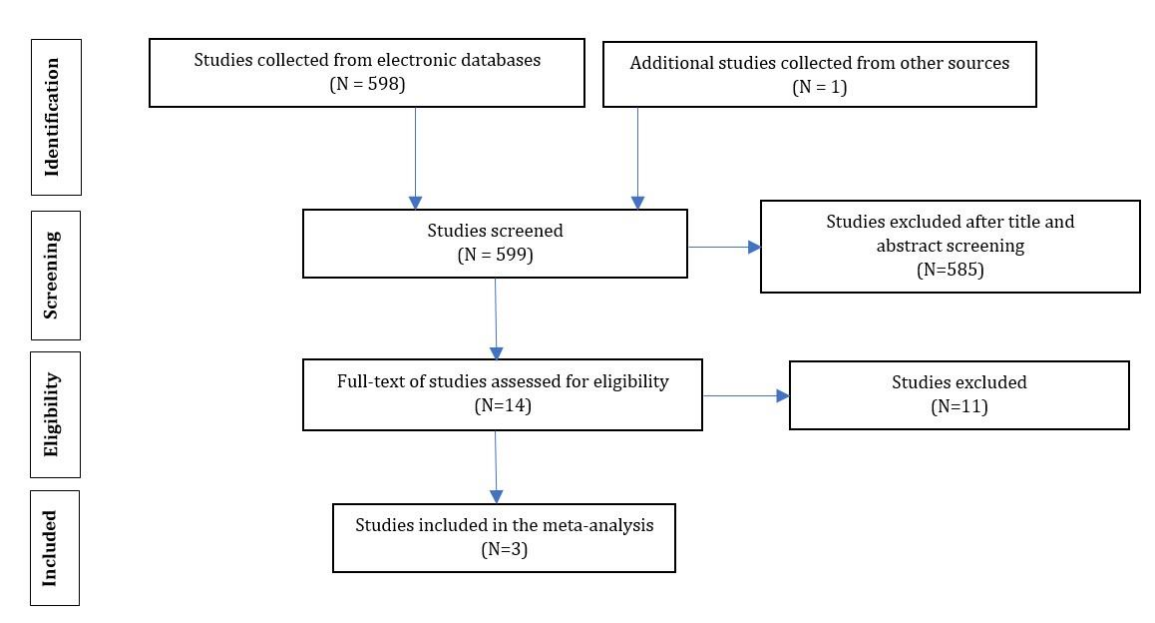
Flowchart of the study and selecting the articles based on the PRISMA steps

**Figure 2:**
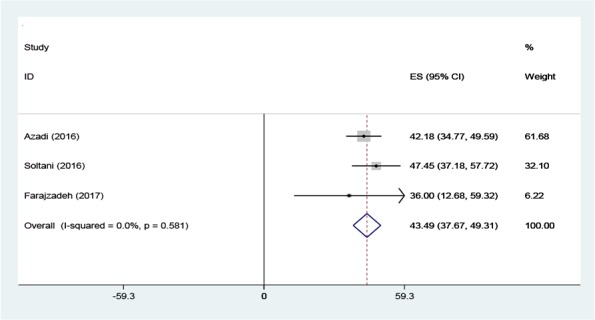
The Forest plot of the overall and individual matched mean scores of knowledge with a 95% confidence interval

**Figure 3:**
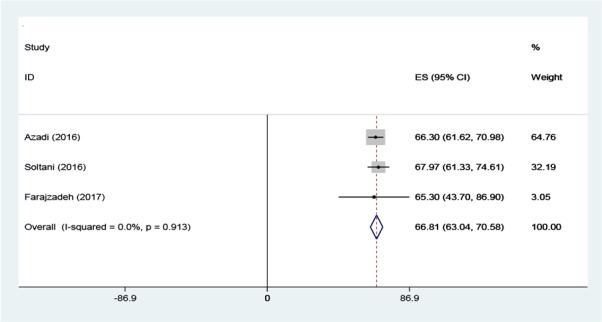
The Forest plot of the overall and individual matched mean scores of attitude with a 95% confidence interval

**Figure 4:**
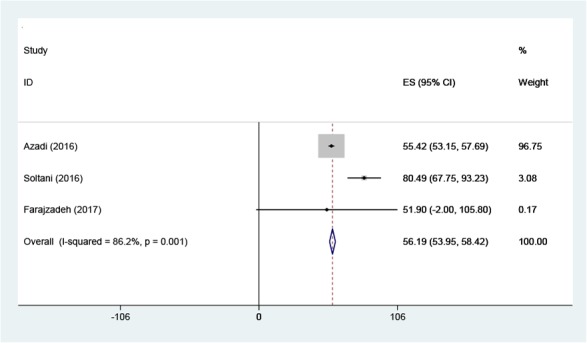
The Forest plot of the overall and individual matched mean scores of performance with a 95% confidence interval

**Figure 5:**
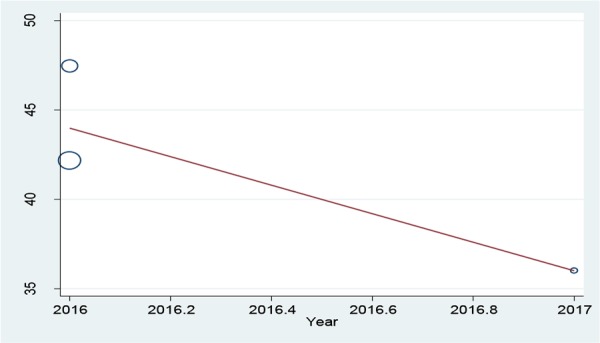
The meta-regression analysis of the mean scores of knowledge versus the year of study

**Figure 6:**
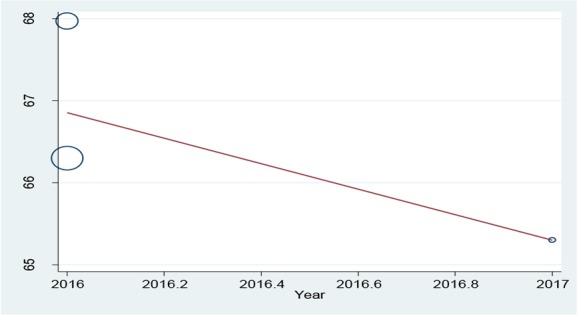
The meta-regression analysis of the mean scores of attitude versus the year of study

**Figure 7:**
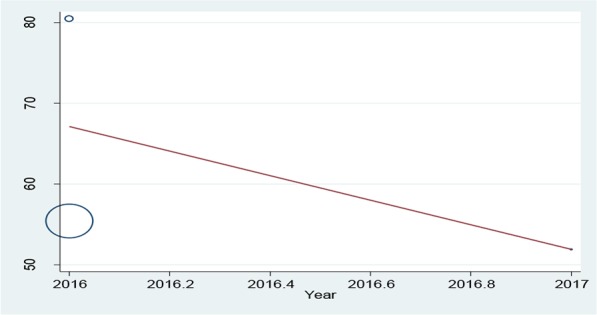
The meta-regression analysis of the mean scores of performance versus the year of study

The initial search performed in the present study retrieved 599 articles, three of which were selected as eligible for the meta-analysis (figure 1). The total number of participants in the analysis was 586, including 397 women and 189 men (table 1). All the included studies had a cross-sectional design. All the included studies had reported the levels of knowledge, attitude and performance associated with disaster preparedness in nurses in different hospitals in Iran. Before the statistical analysis, matching was performed based on the maximum mean scores reported in a study as 80 for knowledge and attitude and 100 for performance (table 2). After the matching, the knowledge score was categorized as poor (0–26), average (27–53) and high (54–80) and attitude scores was categorized as poor (20–40), average (41–60) and high (61–80). The performance score was also categorized as weak (0–33), average (34–67) and high (68–100). According to figures 2–3 and 4, the mean scores of the knowledge, attitude and performance of the nurses associated with disaster preparedness were respectively calculated as 43.49 (95% CI: 37.67–49.31, P=0.581, I^2^=0.0%), 68.81 (95% CI: 63.04–70.58, p=0.913, I^2^=0.0%) and 56.19 (95% CI: 53.95–58.42, P=0.001, I^2^=86.2%). The present study found the heterogeneity between the knowledge scores and also between attitude scores to be low and that of the performance scores to be high. Figures 5–7 show the results of meta-regression analysis by the year of study. Forest plots were produced for overall and individual matched mean scores for each study using the fixed effects model. The lines represent the confidence interval and the midpoint of each line shows the mean value in each study. The diamond represents the overall mean confidence interval for all the studies.

**Table 1:** Characteristics of the included articles

**First author**	**City**	**Publication year**	**Sample size**	**Number of females**	**Number of males**	**Mean age**	**Sampling method**
Azadi (1)	Ilam	2016	112	56	56	32.5±6.08	census
Soltani (2)	Yazd	2018	217	170	47	33.94±6.4	random
Faraj zadeh (4)	Saghez	2017	257	171	86	33.9±7.3	census

**Table 2:** Matched and mismatched mean scores

**First author**	**Knowledge (Mismatched)**	**Knowledge (Matched)**	**Attitude (Mismatched)**	**Attitude (Matched)**	**Performance (Mismatched)**	**Performance (Matched)**
Azadi	9.5±3.78	42.18	27.35±2.39	66.30	3.88±7.33	55.42
Soltani	13.05±5.24	47.45	28.94±3.39	67.97	45.88±6.5	80.49
Farajzadeh	36±11.9	36.00	65.3±11.02	65.30	51.9±27.5	51.90

## Discussion

The present study observed moderate levels of knowledge and performance and high levels of attitude associated with disaster preparedness in Iranian nurses. Nurses’ inadequate knowledge of disaster preparedness is associated with the authorities’ failure to hold efficient training programs during their educational courses and working periods in hospitals. This deficiency is recommended to be resolved by paying a special attention to nurses as the largest group of healthcare providers with a key role in alleviating the harmful health consequences of disasters (11). Ibrahim et al reported acceptable attitudes and a lack of knowledge about disaster preparedness and poor performance in the study nurses (12). Educational systems in Iran and many other countries are not adequately developed to address the needs of nurses for disaster preparedness, leaving nurses undernourished in terms of knowledge and skills in disaster management (13). Ghanbari et al found low levels of disaster preparedness in nurses, and recommended implementing training programs to improve their preparedness for disasters (14). Research also suggests low levels of knowledge in other healthcare staff in hospitals, including medical record personnel, potentially due to a lack of planning and failing to implement disaster preparedness policies in hospitals (15).

Healthcare personnel were, however, found to have positive attitudes toward disaster preparedness, and therefore were willing to participate in disaster preparation programs. Providing these personnel with systematic and regular training is therefore essential for increasing their knowledge and performance during both their education and working periods. The present study results suggested a declining trend in the levels of knowledge, attitude and performance associated with disaster preparedness in the nurses, which can be explained by their lack of motivation to participate in disaster preparation programs probably due to their being overwhelmed by workload. In addition, the fatigue associated with heavy workloads can inhibit nurses from involving themselves in more difficult tasks. The committee of emergencies and disasters in hospitals is recommended to monitor the educational needs by holding regular meetings and implementing training programs for all hospital personnel according to accreditation measures. Annual exercises and drills should also be performed in hospitals to implement the learned practices and identify and resolve the deficiencies and weaknesses using complementary training programs.

### Limitations

The number of studies conducted on knowledge, attitude and performance associated with disaster preparedness in nurses was limited. Different studies had reported different mean scores for knowledge, attitude and performance using different data collection tools. The heterogeneity was resolved using the scores reported in one of the studies.

### Recommendations

As the largest health provider group, nurses play a vital role in responding to disasters. To empower nurses and other hospital staff against disasters, preparation programs are recommended to be standardized using educational packages, hospital incident command systems (HICS) to be established, accreditation instructions to be developed and these standards to be exercised in hospital settings.

## Conclusion

Knowledge and performance associated with disaster preparedness were moderate and attitude was good in Iranian nurses. Teaching nurses and implementing training programs such as hospital exercises appear helpful in promoting the overall preparedness of nurses for disasters.
